# Clinical oral dryness score: evaluation of a new screening method for oral dryness

**DOI:** 10.1007/s10266-018-0339-4

**Published:** 2018-01-22

**Authors:** Derk H. Jan Jager, Casper P. Bots, Tim Forouzanfar, Henk S. Brand

**Affiliations:** 1 Department of Maxillofacial Surgery and Oral Pathology, VU University Medical Center, Amsterdam Movement Sciences, Boelelaan 1118, PO Box 7057, 1007 MB Amsterdam, The Netherlands; 2 Department of Oral Health Sciences, KU Leuven & University Hospitals Leuven, Kapucijnenvoer 7, 3000 Leuven, Belgium; 3 Dutch Institute for Salivary Research, Tuinfluiter 5, 3752 NA Bunschoten, The Netherlands; 4 Department of Oral Biochemistry, Academic Center for Dentistry Amsterdam (ACTA), Gustav Mahlerlaan 3004, 1081 LA Amsterdam, The Netherlands

**Keywords:** Hyposalivation, Xerostomia, Saliva, Screening, Indexes, Mouth

## Abstract

The purpose of this study was to explore the association of the clinical oral dryness score (CODS) with salivary flow rates, xerostomia inventory (XI), and bother index (BI). 147 patients were screened using CODS, which determined 10 features of oral dryness. Each feature contributed 1 point, and the total score varied from 0 to 10. Unstimulated (UWS), chewing-stimulated (CH-SWS) and acid-stimulated (A-SWS) whole salivary flows and the XI and BI were measured. Associations were explored with a bootstrapped Spearman rank correlation test (1000 × bootstrapping). Based on unstimulated salivary flow, 55 patients were classified as hyposalivators, 31 as low salivators, 48 as normosalivators and 13 as high salivators. Median CODS in the hyposalivation group was 5 (IQR 3–6) compared with 3 (IQR 2–5) in the low salivation group, 2 (IQR 1–4) in the normal salivation group and 2 (IQR 1–2.5) in the high salivation group. Significant associations between CODS and the other parameters were only found in the hyposalivation group between CODS and UWS (*ρ*(53) = − 0.513; *p* < 0.01), between CODS and CH-SWS (*ρ*(53) = − 0.453; *p* < 0.01), between CODS and A-SWS (*ρ*(53) = − 0.500; *p* < 0.01), CODS and XI (*ρ*(53) = 0.343; *p* < 0.001) and between CODS and BI (*ρ*(53) = 0.375; *p* = 0.01). In patients with hyposalivation, CODS is associated with unstimulated and stimulated salivary flow and XI and BI. CODS alone or a combination of CODS with a subjective measure, such as the XI or BI, could be recommended during routine clinical assessment to detect hyposalivation.

## Introduction

Whole saliva is a complex oral fluid comprising a mixture of secretions from major and minor salivary glands, with additional contributions by crevicular fluid [1]. Saliva plays a pivotal role in preserving the integrity of the oral environment, via lubrication and protection [2]. Nevertheless, it is hard to determine precisely when diminished saliva secretion results in oral problems such as an experience of dry mouth (xerostomia) [3]. However, it has been demonstrated that a diminished salivary flow rate is associated with mucosal changes, and signs and symptoms may emerge [4]. An accurate quantification of the salivary flow would require each patient to be followed longitudinally [3], as the amount of saliva is subject to intra-individual differences [2]. Considering the impact of a decreased salivary flow rate on a person’s quality of life and oral health, it has been suggested that assessment of salivary function should be part of routine dental check-ups [3, 5]. In the general dental practice, quantification of the unstimulated, chewing-stimulated and citric acid-stimulated saliva secretion rates is hardly performed as many dentists consider it time consuming and not part of their daily routine.

In addition to objective quantification of the saliva secretion, several subjective measures are available to assess the symptoms and burden for patients experiencing dry mouth. The xerostomia inventory (XI) is a validated and frequently used questionnaire, that explores how the patient experiences dry mouth (xerostomia) [6]. This questionnaire consists of 11 items, each on a 5-point Likert scale. Another self-reported index is the Bother Index (BI). In the BI, the patient is asked to rate the severity of dry mouth on a scale from 0 to 10 [7]. Both the XI and the BI are based on self-report, and are therefore subjective. Recently, a new instrument was designed to objectively quantify clinical signs of reduced salivary secretion: the clinical oral dryness score (CODS). The aim of the CODS is to provide a quick, easy and objective method to determine salivary gland function in the clinical setting, such as a dental practice or maxillofacial outpatient’s clinic. The CODS is designed to assess oral dryness by clinical and visual inspection of the oral cavity based on several signs of oral dryness such as the presence of frothy saliva, the dryness of the oral mucosa and stickiness of the dental mirror to the tongue or the buccal fold [8]. In the study by Osailan, it was suggested that the CODS is a reliable routine assessment of the severity of hyposalivation.

Until now, the CODS has not been reviewed and evaluated in more detail. In this study it is hypothesized that the CODS is associated with salivary flow rates and the aim of this retrospective case series study was to investigate the association of the CODS with salivary flow rates among a heterogeneous group of patients. Furthermore, we investigated whether there is a relationship between the CODS and xerostomia measured by two subjective measures (XI and BI).

## Materials and methods

### Study design

A retrospective case series study was designed. Data were collected through convenience sampling from 147 patients (mean age, 53 ± 17 years; female to male ratio, 1.5:1) who attended the saliva clinic of the Centre for Special Care Dentistry Amsterdam, the Netherlands, between December 2011 and December 2015. Dentists, physicians, or medical specialists referred patients to the saliva clinic. Patients were included in this study when all questionnaires and all data, necessary for this study, were present in their patient record. The heterogeneous study population comprised patients who could be stratified into five groups based on their reason for referral: (1) dental or restorative problems such as dental erosion, wear, bruxism and caries (50 patients); (2) medication-related hyposalivation and/or xerostomia (34 patients); (3) Sjögren’s syndrome (27 patients of which 10 patients with a secondary syndrome and 17 with a primary syndrome (according to AECG criteria) [9]); (4) radiotherapy in head and/or neck region (3 patients); 5) control group (33 patients; no reported xerostomia/hyposalivation). The patient population was also stratified according their unstimulated whole mouth salivary (UWS) flow into four groups: hyposalivation (UWS ≤ 0.1 mL/min, 55 patients), low salivation (UWS > 0.1–0.2 mL/min, 31 patients), normal salivation (UWS > 0.2–0.5 mL/min, 48 patients) and high salivation (UWS > 0.5 mL/min, 13 patients). This study followed the Declaration of Helsinki on medical protocol and ethics. The experiment was performed in accordance with the guidelines of the Ethics Review Committee of the VU University Medical Center. The Ethics Review Committee of the VU University Amsterdam confirmed that the Medical Research Involving Human Subjects Act (WMO) does not apply to this study. Written informed consent was obtained of each patient. The reporting of this study conforms to the STROBE statement [10].

### Data collection methods

Case report forms (CRFs) were designed to collect data in a standardized manner. One data abstractor, with specialized knowledge of the research question (DHJJ), performed data abstraction from the medical charts to the case report forms. To verify incorrect transfer of data from the medical record to the CRF, random checks were performed prior to data entry. This was done according to the 100–20 rule in which 100% of the data is checked in 20% of the CRFs and 20% of the most important data was checked in 100% of the CRFs to prevent mistakes in data retrieval [11].

### Variables

#### Subjective oral dryness

Before a patient visited the clinic he or she received questionnaires on paper by mail to fill out at home. The first questionnaire was the XI [12]. The XI consists of 11 statements about mouth feel and oral dryness, each with a 5-point Likert scale to indicate how often the patient suffers from problems with regard to mouth feel and oral dryness. The scores from the 11 questions are summed resulting in a total XI score that varies from 11 (no dry mouth) to 55 (extremely dry mouth). The second questionnaire was the BI. This index quantifies the burden related to the dry mouth on a 10-point scale. Scores range from 0 to 10, where 0 is no burden and 10 is excruciating burden [7].

#### Clinical oral dryness score

The CODS used in the present study consisted of a 10-point scale; each point representing a feature of dryness in the mouth as follows: (1) mirror sticks to buccal mucosa. (2) Mirror sticks to tongue. (3) Tongue lobulated/fissured. (4) Tongue shows loss of papillae. (5) Frothy saliva. (6) No saliva pooling in floor of mouth. (7) Glassy appearance of other oral mucosa, especially palate. (8) Debris on palate (excluding debris under dentures). (9) Altered/smooth gingival architecture. (10) Active or recently restored (last 6 months) cervical caries (> 2 teeth) [8]. The scores from the ten features were added together resulting in a total CODS. A high total score indicates increased severe oral dryness.

Every patient was examined by one examiner (DHJJ or CPB). The examiner scored the features observed in the patient’s mouth, using a specially designed form with illustrations of dry mouth features. This form used pictures from the original publication describing the CODS [8]. Before collecting salivary flow data and analysing the xerostomia questionnaires, the examiners first recorded the CODS. This way, the examiners were not aware during the recording of the CODS whether a patient was a hyposalivation/xerostomia or a non-hyposalivation/non-xerostomia patient.

#### Sialometry

Unstimulated whole saliva (UWS), chewing-stimulated whole saliva (CH-SWS), and citric acid-stimulated saliva (A-SWS) were collected in a standardized manner. Patients were instructed to refrain from eating, drinking, chewing gum, brushing teeth, using mouthwash, and smoking for 60 min prior to visiting the clinic. All assessments were made between 8:00 am and 12:00 pm to minimize fluctuations associated with the circadian rhythm of salivary secretion [13].

At the time of visit, each patient was placed in a quiet room and asked to sit in an upright position. Unstimulated saliva was collected by the draining method in a pre-weighed plastic container [5]. Patients were instructed to begin collecting saliva immediately after an initial swallow and to expectorate in the container as soon as they collected saliva. During the collection period (5 min) patients were not allowed to swallow. To collect SWS, patients were asked to chew a 5 × 5 cm sheet of paraffin (Parafilm M, Pechiney, Chicago, IL, USA) at a chewing frequency of approximately 60 chews per minute and expectorate into a pre-weighed container every 30 s during a 5 min period. For A-SWS, saliva secretion was stimulated with a citric acid solution (2% w/v) applied with a cotton wool swab to the lateral borders of the tongue at 30-s intervals [14]. After the collection period the plastic containers were reweighed and the collected volume was determined by subtracting the weight of the container prior to collection. Salivary flow was calculated by dividing the collected volume (assuming 1 g of saliva equals 1 mL) with collection time (min) and values were expressed in mL/min [5].

### Data analysis

Data were assessed for normality using the Shapiro–Wilk test. As all parameters were not normally distributed, the data are presented as median and their interquartile range (IQR). Differences between CODS of the salivation subgroups were examined using the Mann–Whitney *U* test (significance level was set at 0.05). Possible relationships between XI, BI, CODS, UWS, A-SWS and CH-SWS were explored with a bootstrapped Spearman rank correlation test (1000 × bootstrapping). Data were analysed using SPSS, version 22.0 (IBM Corp, Armonk, NY, USA). A significance level (*α*) of 0.01 was chosen for the correlation test.

## Results

The UWS, CH-SWS, A-SWS, BI, XI, and CODS data were not normally distributed (Shapiro–Wilk test; *p* < 0.01). Analysis of patients according to reason for referral revealed that the lowest median UWS values were found in the Sjögren’s syndrome group (*Mdn* = 0.02 mL/min, IQR 0.0–0.6) and the highest median UWS values were found in the control group (*Mdn* = 0.22 mL/min, IQR 0.09–0.35) and in the erosion, wear, bruxism, caries (EWBC) group (*Mdn* = 0.26 mL/min, IQR 0.18–0.36).

The patients were also grouped according to their UWS flow rates into hyposalivation, low, normal and high salivation groups. The median UWS, CH-SWS, A-SWS and CODS values for each of these subgroups and the overall study population are presented in Table 1. There was a significant difference in CODS between the hyposalivation (*Mdn* = 5, IQR 3–6) and the low salivation group (*Mdn* = 3, IQR 2–5; *U* = 520,5, *p* = 0.003, *r* = − 0.33), the normal salivation group (*Mdn* = 2, IQR 1–4; *U* = 470 *p* < 0.01, *r* = − 0.56) and the high salivation group (*Mdn* = 2, IQR 1–2.5; *U* = 71, *p* < 0.01, *r* = − 0.55). Also, there was a significant difference between the low salivation group and the normal salivation group (*U* = 517.5, *p* = 0.02, *r* = − 0.26), and the high salivation group (*U* = 97, *p* = 0.006, *r* = − 0.41). The normal and high salivation groups did not differ significantly (*p* = 0.27).

**Table 1:** The median UWS, CH-SWS, A-SWS, XI, BI and CODS and their corresponding IQRs for the overall group and the four subgroups based on their salivation: hyposalivation, low, normal and high salivation

	UWS	IQR	CH-SWS	IQR	A-SWS	IQR	XI	IQR	BI	IQR	CODS	IQR
Hyposalivation	0.02^*^	0.0–0.06	0.85^&^	0.35–1.45	0.22^$^	0.06–0.42	39^#^	35–45	8^@^	5–9	5^*,&,$,#,@^	3–6
Low salivation	0.14	0.12–0.18	2.2	1.3–2.75	0.76	0.44–1.06	25	19–38	6	2–8	3	2–5
Normal salivation	0.3	0.26–0.39	2.45	1.63–3.39	1.13	0.76–1.41	22.5	17.25–30	3	1–7	2	1–4
High salivation	0.66	0.56–0.92	3.3	2.63–4.4	2.02	1.28–2.62	28	17.5–42	4	0.5–7.5	2	1–2.5
Overall	0.16^*^	0.04–0.3	1.9^&^	1.05–2.85	0.7^$^	0.37–1.21	28^#^	20–39	6^@^	2–8	3^*,&,$,#,@^	2–5

Significant (*p* < 0.01) associations between CODS and the other parameters, within each group, are marked with corresponding symbols

Table 2 shows how frequently each item of the CODS was scored. In the overall study population, item 1 (the mirror sticks to the cheek; 21%) was most frequently scored, and item 8 (food debris on the palate; 1%) the least. When stratified according to the UWS, item 1 was most frequently scored in the normal salivation group and least frequently in the hyposalivation group. Item 4 (fissured tongue) was most frequently scored in the hyposalivation group and least frequently in the low salivation group. Item 4 was scored more frequently in the normal salivation group compared with the low salivation group. In the high salivation group three items related to severe dry-mouth complaints were not scored at all, including item 6 (no saliva pooling in floor of mouth).

**Table 2:** Analysis of separate CODS items

CODS item	Overall *n* = 147	UWS < 0.1 *n* = 55	UWS 0.1–0.2 *n* = 31	UWS 0.2–0.5 *n* = 48	UWS > 0.5 *n* = 13
	*Mdn* Cods = 3 (%)	*Mdn* Cods = 5 (%)	*Mdn* Cods = 3 (%)	*Mdn* = 2 (%)	*Mdn* Cods = 2 (%)
(1). Mirror sticks to buccal mucosa	22.2	18.3	23.9	28.8	25
(2). Mirror sticks to tongue	15	14.5	16.5	15.3	12.5
(3). Tongue lobulated/fissured	7.4	6.9	8.3	7.6	8.3
(4). Tongue shows loss of papillae	7.4	8.8	3.7	8.5	4.2
(5). Frothy saliva	12.7	9.5	15.6	13.6	29.2
(6). No saliva pooling in floor of mouth	7.6	12.6	3.7	1.7	0
(7). Glassy appearance of other oral mucosa, especially palate	9.9	10.7	10.1	7.6	12.5
(8). Debris on palate (excluding under dentures)	1	1.15	0.9	0.8	0
(9). Altered/smooth gingival architecture	7.6	7.6	8.3	8.5	0
(10). Active or recently restored (last 6 months) cervical caries (> 2 teeth)	9.4	10.3	9.2	7.6	8.3

The table shows how frequently each CODS item was scored (in %) in the overall group and stratified according to their UWS hyposalivation, low salivation, normal and high salivation groups

When analysing the associations between CODS and the salivary parameters (UWS, CH-SWS, A-SWS), XI and BI, significant associations in the overall study population were found between CODS and UWS (*ρ*(145) = − 0.554; *p* < 0.01; 95% CI − 0.659 to − 0.419), CODS and CH-SWS (*ρ*(145) = − 0.579; *p* < 0.01; 95% CI − 0.666 to − 0.462), CODS and A-SWS (*ρ*(145) = − 0.467; *p* < 0.01; 95% CI − 0.582 to − 0.306), CODS and XI (*r*(145) = 0.343; *p* < 0.01; 95% CI 0.196 to − 0.477) and between CODS and BI (*ρ*(145) = 0.375; *p* < 0.01; 95% CI 0.227 to 0.498). When analysing the patients stratified according to their degree of salivation (hypo-, low, normal or high salivation) significant associations between CODS and the other parameters were only found in the hyposalivation group: between CODS and UWS (*ρ*(53) = − 0.513; *p* < 0.01; 95% CI − 0.704 to − 0.291), between CODS and CH-SWS (*ρ*(53) = − 0.453; *p* < 0.01; 95% CI − 0.625 to − 0.223), between CODS and A-SWS (*ρ*(53) = − 0.500; *p* < 0.01; 95% CI − 0.693 to − 0.277), CODS and XI (*ρ*(53) = 0.343; *p* < 0.001; 95% CI 0.195 to 0.476) and between CODS and BI (*ρ*(53) = 0.375; *p* = 0.01; 95% CI 0.236 to 0.497).

The association between UWS and the subjective, patient-reported severity of dry mouth measures (XI and BI) was also analysed. In the overall study population, a significant association was found between UWS and XI (*ρ*(145) = − 0.380; *p* < 0.01; 95% CI − 0.519 to − 0.221) and between UWS and BI (*ρ*(145) = − 0.365; *p* < 0.01; 95% CI − 0.508 to − 0.210). In addition, the XI and BI correlated significantly with each other (*ρ*(145) = 0.82; *p* < 0.01; 95% CI 0.746 to 0.880). When patients were stratified according to their degree of salivation, only in the hyposalivation group a significant association was observed between UWS and XI [(*ρ*(53) = − 0.478; *p* < 0.01; 95% CI − 0.683 to − 0.251] and between UWS and BI [(*ρ*(53) = − 0.319; *p* = 0.01 95% CI − 0.582 to − 0.260].

All the significant associations can be considered robust to distributional violations as the bootstrapped 95% CIs did not exceed 0.

## Discussion

In the present study we explored the CODS in four subgroups of patients with different degrees of salivation, and we demonstrated that the CODS is related to unstimulated and stimulated salivary flow in patients with hyposalivation.

Compared to the study by Osailan et al. [8], the mean CODS values in the present study were lower in the overall and in the subgroups with different salivary flow-rates. A smaller proportion of severe hyposalivation patients with obvious clinical signs in the study population could explain this difference.

Not unexpectedly, CODS item 6 (a dry floor of the mouth, no saliva present) was scored only positive in the most severe hyposalivation patients. In contrast, some other findings were unexpected. The most surprising finding was that item 1 (mirror sticks to the buccal mucosa of the cheek) was scored most frequently in the normal salivation group, and least frequently in the hyposalivation group (Table 2). This apparent contradiction could be explained by the composition of the normal salivation group. The group contained, next to patients from the control group, patients with dental erosion, dental wear, or medication-related xerostomia. Increased susceptibility to erosion and tooth wear could be related to an altered protein composition of saliva. Specifically, mucins are thought to play an important role in preventing demineralization [15]. Thus, the dental erosion patients in the normal salivation group could have had lower salivary mucin concentrations. Moreover, lower salivary protein concentration, especially of mucins, can result in less lubrication [16, 17] and result in sticking of the mirror to the buccal mucosa of the cheek in patients with normal salivary flow levels. A publication by Chaudhury et al. [18] provides an alternative explanation. They reported that in xerostomia patients the UWS mucin concentration is not changed, but that the glycosylation of the mucins is altered leading to functional impaired mucins. These mucins retain less water and subsequently to a reduced hydration of mucosal surfaces such as the cheek. In support of our findings with respect to item 1 of the CODS, they found that patients with healthy UWS (i.e., > 0.2 mL/min) but complaining of a dry mouth feeling (xerostomia) had lower total mucin/glycan proportion and glycosylation. This indicates a reduction in mucosal hydration and rheological properties even in xerostomia patients with healthy salivary flow rates [18]. This could also apply to patients with medication-related xerostomia in the normal salivation group. Certain classes of drugs can induce hyposalivation or xerostomia by targeting neurotransmitters and receptors. Consequently, the salivary composition can change. Patients with medication-related xerostomia often have a normal salivary flow rate, but with reduced protein concentration. Drugs that inhibit neurotransmitter binding to acinar membrane receptors or that interfere with ion transport pathways may affect the quantity as well as the quality of the saliva [19]. The designers of the CODS were already aware of this possible problem as they also explored the relationship between CODS and mucosal wetness. Patients with similar flow rates appeared to have significantly different mucosal wetness [8]. Collectively, these findings suggest that there may be changes in the coating properties of saliva as a result of differences in saliva protein content or function between patients. Unfortunately, this could not be studied in the present study.

From our data, it appeared that the CODS was only significantly related to UWS in the hyposalivation group. Therefore, in our opinion, the CODS can only be used to differentiate between hyposalivation and normal salivation. However, the score would still be useful in the general clinical setting, as this information is important in the examination of patients. We also found that there was a significant association between the CODS and the subjective, patient-reported severity of dry mouth (XI, BI) in the overall group (Table 1). In addition, the subjective measures correlated significantly with each other and with the UWS in the overall group (Table 2). This corresponds with previous research using the Xerostomia Inventory [20]. When patients were grouped according to their degree of salivation, only the hyposalivation group showed a significant association between XI, BI, and CODS. Furthermore, only the hyposalivation group showed a significant association between the subjective measures (XI, BI) and salivary flow data (Table 1). This suggests that most of the patients in our cohort become aware of dry mouth symptoms when salivary flow drops below 0.1 mL/min. Taken together, our data suggest that the XI, BI and CODS are all able to differentiate between hyposalivation and normal salivation. Therefore, the combination of a subjective measure and the CODS could provide the general practitioner with easy-to-use tools to identify hyposalivation patients.

For the CODS, the designers incorporated several clinical criteria resulting in a total of 10 features with the aim to discriminate between normal, moderate, and severe hyposalivation. The suggested model was a first attempt to design a model to semi-quantitatively assess oral dryness in patients complaining of xerostomia. Our evaluation of the model partly confirms earlier findings described by Osailan et al. [8], but further optimization of in a large cross-section of the population seems desirable.

## Conclusion

This study is the first to use the Clinical Oral Dryness Score in a survey of a large, heterogeneous group of patients. The results indicate that, in patients with hyposalivation, CODS is associated with unstimulated and stimulated salivary flow and XI and BI. A combination of the CODS with a subjective measure, such as the XI or BI, into routine clinical assessment of patients with dry mouth complaints in dental practices or maxillofacial surgery clinics could be recommended and is easy to perform. Further research is required to investigate whether optimization of the model and better discrimination between hyposalivation and normal salivation is possible with this score.
